# Perceptions of Behavioral Awareness, Intention, and Readiness for Advance Care Planning: A Mixed-Method Study among Older Indigenous Patients with Late-Stage Cancers in Remote Areas of Eastern Taiwan

**DOI:** 10.3390/ijerph18168665

**Published:** 2021-08-17

**Authors:** In-Fun Li, Sheng-Miauh Huang, Ching-Fang Lee, Yi-Heng Chen, Yvonne Hsiung

**Affiliations:** 1 Department of Nursing, Tamsui Branch, Mackay Memorial Hospital, New Taipei City 25160, Taiwan; lif1129@mmh.org.tw; 2 Department of Nursing, MacKay Medical College, New Taipei City 25245, Taiwan; r910862@mmc.edu.tw (S.-M.H.); chingfang@mmc.edu.tw (C.-F.L.); yiheng@mmc.edu.tw (Y.-H.C.)

**Keywords:** advance care planning, behavioral readiness, indigenous patients, late-stage cancers, Taiwan

## Abstract

The first Patient Right to Autonomy Act enacted in Asia in 2019 has enabled every Taiwanese citizen to plan for his/her end-of-life (EOL) in case of incompetency. Advance care planning (ACP) has been highly promoted for individuals with terminal, life-threatening illnesses, particularly in the mainstream society, and efforts have been made by the Taiwanese government to train health care providers in order to optimize patients’ quality of dying. However, such advanced decisions and discussions regarding life-sustaining treatment and EOL care remain scarce among older ethnically minority patients. A multiple-case study employing a mixed-method (*n* = 9) was undertaken to explore indigenous patients’ ACP perceptions. Both quantitative and qualitative information was obtained from indigenous patients, a minority group whose socio-economic and educational status are different from the general Taiwanese population. An initiative was made to describe ACP behavioral awareness, intention, and readiness of older terminal patients from four tribes with seven late-stage cancers in remote, mountainous areas of eastern Taiwan. Our findings showed that according to the Transtheoretical Model, terminal indigenous patients’ ACP readiness was at a precontemplation stage. Their lack of fundamental ACP awareness, insufficient healthcare resources, life-sustaining value in a Christian faith context, and the prevalent health disparity in the remote communities have negatively affected indigenous patients’ intention to participate in ACP. We provide suggestions to further promote ACP in this group and suggest that health information should be tailored at various readiness stages in order to overcome barriers and decrease ACP literacy discrepancies. This study calls attention to an understudied area of ACP behaviors, an overlooked need in EOL care for older cancer patients of unique cultural backgrounds, and the imperativeness to ensure cultural minority group’s EOL care is consistent with patients’ preferences.

## 1. Introduction

In the past two decades, governmental and societal efforts were made in Taiwan to promote advance care planning (ACP), the communication process documented to ultimately assure patient autonomy and protect human dignity, and the first Asian Patient Right to Autonomy Act [1] enacted in 2019 has enabled every Taiwanese citizen to plan for his/her end-of-life (EOL) in case of incompetency. Health care providers are particularly trained to provide patient autonomy counseling regarding life-sustaining treatment and EOL care to those of limited life expectancy [2]. Advanced treatment and care decision-making has proven to effectively assist terminal patients to increase a sense of self-control, alleviate sufferings, and facilitate peaceful deaths which are particularly consistent with older patients’ desires [3,4]. Previous Taiwanese studies among community-residing older patients show that not only financial burdens but also psychological encumbrances related to difficult decision-making could be minimized by communicating EOL preferences with loved ones [5,6,7]. However, although ACP has been particularly encouraged for older patients with terminal and life-threatening illness [8,9], research published relevant to older Taiwanese patients’ EOL decision-making were mostly related to advance directives (ADs) completions and life-support utilization [10,11,12]. Existing literature has been based almost entirely on the mainstream Taiwanese group (Han tribe) members under 65-years-old in urban settings [13]. In addition, discussions and documentations of life-sustaining treatment and artificial nutrition and hydration remained scarce among older minority patients with life-threatening illnesses in Taiwan [14].

Accounting only for 2.3% of the overall population, there are approximately 530,000 Taiwanese citizens belonging to 16 indigenous tribes [15]. The majority of Taiwanese indigenous peoples dwell away from metropolitan cities. More than half of these groups reside in rural townships or remote mountain areas whereas a large portion of older women live in isolated difficult-to-reach communities, [15]. A health disparity exists in that, over the past 30 years, indigenous people had a nearly 70% higher mortality rate than the rest of the general Taiwanese population [16]. After adjusting for age and gender, indigenous people’s cancer prevalence has annually increased to approximately 20% [15]. Previous national studies showed evident disparities among Taiwanese indigenous residents in mountainous communities who were socio-economically disadvantaged with compromised health access [17]. Compared with the Taiwanese in mainstream society, indigenous peoples were noticeably highly vulnerable to multiple social welfare problems associated with poverty, substance abuse, alcoholism, low literacy, and low life expectancy [18,19,20].

In remote areas of Taiwan, itinerant clinic hours are regularly provided to serve villages with population under 1000–2000 within 10 miles, such as rural areas of Taitung and Hualian provinces. Local oncologists in indigenous peoples aggregations are normally referred by itinerant physicians so that oncology patients may receive standard cancer care under the national health insurance. It is extremely unlikely that indigenous patients with a terminal illness condition of late-stage cancer have not utilized any health service in Taiwan, unless they have never yet been diagnosed with cancer. Such health access to itinerant services, however, is incomparable to the abundant options of cancer care in the mainstream society, and huge rural–urban gaps evidently exist regarding the quality of health access and resources. In addition, common inequity issues are mostly complicated by limited transportation in rural or remote areas—certain types of (self-paid) medicines, availability of surgical procedures, counselling services, and facilities to provide palliative care often fall short in remote areas. The recently enacted ACP is still not a well-received concept among the general public and generally discussed in teaching hospitals of metropolitan cities; even in large suburban hospitals, ACP is not proactively discussed with terminal patients by health care providers; not to mention the average ACP uptake is low among ethnic minority groups, mainly due to patients’ unawareness and language barriers [5]. If ACP was to be discussed and implemented, indigenous patients with terminal illnesses could be equipped with the knowledge necessary to make their own EOL decisions. In rural or remote areas of Taiwan, ACP discussions and the following execution of EOL preferences in the advance directives still require further assistance from the health care system [18].

Research showed that unawareness of palliative care and ACP related concepts has led to relatively greater helpless and less EOL decision-making participation in minority patients, compared to their counterparts in the mainstream society [21,22,23]. Indigenous patients with late-stage cancer would anticipate encountering ACP inequality issues when health care providers attempt to deliver relatively complex health information without using indigenous languages. According to the original Transtheoretical Model (TTM) [24,25], individuals move through various stages of behavioral change: precontemplation, contemplation, preparation, action, maintenance. In the TTM, awareness and intention serve as precursors of actual behavioral changes, and precontemplators, who have the lowest behavioral readiness, usually do not intend to take action in the foreseeable future (defined as within the next 6 months); they are often unaware of their problematic behavior which produces negative consequences. Consciousness raising, increasing awareness about the healthy behavior, is believed to be the most important and effective strategy to facilitate precontemplators transiting to the next stage of change, contemplators. In our case, the TTM may provide theoretical guidance to assess and increase older indigenous patients’ behavioral readiness for ACP.

To our knowledge, no prior study has assessed Taiwanese indigenous patients’ ACP, in particular their awareness, intention, and behavioral readiness to participate in EOL decisions [24,25]. Due to the scarce literature on the subject [26,27], the way in which ACP is currently perceived and delivered to indigenous patients by their health care providers in remote communities is still unknown. An exploratory multiple-case study design [28,29] was chosen to increase the credibility and validity of research findings by combining both qualitative and quantitative methods [30]. A community initiative was made to understand ACP among underserved, indigenous patients in remote, mountainous areas of eastern Taiwan. Conducted from January to September 2017, this mixed-method study was by far the first multi-case study [31,32] that depicted ethnic minority patients’ complex ACP decision-making behavior.

## 2. Materials and Methods

### 2.1. A Case Study Approach

A case study approach, the exploration of several distinct cases, is usually designed to gain a more in-depth insight into the complex and broad phenomenon among difficult-to-reach cases [33]. To depict ACP decision-making from a group of ethnically diverse patients in remote areas of eastern Taiwan, our study incorporated more than one type of data [28]. While recognizing various realities in the natural setting, several similar cases were viewed as one entity so that assertions might be sought which were common to the whole [33,34]. This multiple-case approach allowed the research team to answer important study questions regarding the existing end-of-life phenomenon in minority cases from a collective viewpoint: Are older, indigenous patients with late-stage cancers in remote areas aware of the ACP option, a national emphasis of health promotion? How behaviorally ready are these terminal patients to contemplate and execute their own ADs as a health means? Do they intend to communicate with their loved ones about life-sustaining treatment and EOL care decisions? Are there other factors that influence older indigenous patients’ ACP readiness?

In this multiple-case study, attention was brought towards terminal minority group’s perceptions of awareness and their intention, especially in relation to the various stages of behavioral readiness for ACP within the Taiwanese mainstream care context. With this in mind, older indigenous participants with late-stage cancers were approached by local research members. Careful attention was paid to cases’ demographic characteristics, such as age, gender, cancer diagnosis, socio-economic and education status, and religious beliefs that previously were shown to be influential in ACP research. When addressing the holistic nature of EOL cancer care [29], a multi-case research approach is particularly beneficial for an unexplored phenomenon in the underserved minority group [28].

### 2.2. Subject Recruiment

Our cases were referred by local oncologists from three indigenous clinics of a community teaching hospital in eastern Taiwan. Potential participants were limited to self-identified indigenous patients belonging to one of the sixteen Taiwanese aboriginal tribes who resided in difficult-to-reach, remote or inaccessible rural villages (population <2000 within 10 miles) [35] of Taitung Province and Hualian Province. The following inclusion criteria for subjects were made clear to the recruiting staff in participating oncology clinics: (1) patients must be aged 70 years or older; (2) diagnosed with at least one Stage III & IV cancer (metastasis to the lymph nodes or other body parts); (3) able to communicate in Mandarin or Taiwanese concerning their current medical conditions and future EOL preferences; (4) legally competent for making decisions regarding life-support and ACP; and (5) resident in the sampled aboriginal aggregations in eastern Taiwan for at least three years to ensure that they were familiar with unique cultural and health needs and issues related to health access in their own neighborhoods.

In a multi-case study design, the research quality lies in obtaining a representative sample. Within the eight months of data collection, 18 potential indigenous patients agreed to participate in this study and none of these potential community residents reported being offended by the research topic. However, nine among them were excluded due to their unavailability to participate in the subsequent in-depth interviews. The drop-out reasons included: deterioration of physical conditions (*n* = 3), lack of transportation to the interview site (*n* = 2), uncertain information regarding cancer stage (*n* = 1), moderate to severe language barriers to discuss ACP (*n* = 2), and unreachability (possible death) (*n* = 1). Our final sample involved nine older indigenous patients from five tribes who were diagnosed with seven types of late-stage cancers with a variant range of socio-demographic and clinical characteristics (Table 1). Specific attention was paid to the influence of gender, yet no evident patterns or noticeable gender difference was found in our final sample.

### 2.3. Data Collection and Data Analysis

Both quantitative and qualitative research methods employed in our multi-case study were based on the constructs of Prochaska’s Transtheoretical Model (TTM) [24,25] for the purpose of exploring minority cases’ behavioral readiness for ACP. In multiple-case studies, data are usually collected from several sources for each individual case [34], and in this present study three types of data were incorporated, including: cancer related information in medical charts, nurse-led semi-structured interviews, and a self-report questionnaire.

A self-administered questionnaire [5,36] was used in which survey questions were derived from previous research among older Chinese ethnic groups with chronic illnesses in the community settings. The questionnaire was originally developed based on the TTM stipulations and literature results of cancer patients’ perspectives regarding their preparedness for EOL care [37,38] to assess an individual’s awareness, intention to change, and behavioral readiness for ACP. Comprised of a series of demographical questions, a measure of behavioral readiness, and a 7-item algorithm of stage of change [39,40], this tool has been modified under the Taiwanese legal and medical context [5]. To date, more than 1400 Taiwanese patients from different clinical settings have used this readiness measure and the ACP algorithm. Older patients using traditional Chinese have reported good clarity and ease of use. In a previous correlational study among community-residing older Taiwanese with chronic illnesses (*n* = 52), subjects were able to complete the whole questionnaire within 15 min and the algorithm served well in distinguishing older patients’ readiness in one of the six stages for their ACP engagement. Good cross-cultural internal consistency was reported among older Taiwanese in northern Taiwan (Cronbach’s alpha = 0.92, *p* <0.01), slightly higher than it was in the original Chinese immigrant population (Cronbach’s alpha = 0.86, *p* < 0.01) [5]. No criterion validity was available.

In this study, considering the limited stamina of terminal patients, only demographic questions and questions in the domains of awareness/knowledge, intention, and behavioral engagement were given. All cases were categorized, based on the algorithm, as ACP precontemplators. Their actual responses to survey questions are shown in Table 2 and Table 3, a typical way to present quantitative results in a multiple-case study (*n* = 9). All survey data that depicted cases’ understanding, willingness or intention to change, and readiness for ACP were managed by SPSS Statistics, Version 19.0 for Macs (IBM Corp, Armonk, NY, USA), and descriptive statistics were used for cases’ rationales behind their ACP decision-making.

A 20- to 30-min face-to-face interview, based on a semi-structured guide [37,38], was scheduled at each older indigenous patient’s convenience to clarify, augment, and corroborate his/her survey responses. The interview guide was specifically informed by relevant experience in interviewing ethnic minority patients with cultural and EOL sensitivities towards ACP awareness and life-support options [5,34,39,40,41]. It was developed from available literature on behavioral readiness and previous studies in relation to Taiwanese indigenous patients’ literacy to ensure relevance to health behaviors of this cultural group [7,14,42,43,44]. The final semi-structured guide was assessed by a group of ACP content experts, including: a cancer nurse specialist of an indigenous background, a local hospice administrator, two nursing professors with palliative care expertise and cultural experience serving in the indigenous areas, and a clinical psychologist specialized in indigenous people’s EOL counseling.

The in-depth interviews were undertaken by the primary investigator mostly in the conference room in the oncology ward after clinic visits. It was first piloted to interview a healthy indigenous individual with chronic illnesses and an older indigenous patient with a late-stage breast cancer; minor modifications were made after the pilot interviews. During the interviews, our interviewers focused on clarifying interviewees’ survey answers and further explored three major topics: indigenous patients’ ACP awareness, intention to change, and behavioral readiness—their ACP engagement. Our cases were encouraged to provide socio-cultural, knowledge, spiritual, and demographic factors that they believed essential to influence their self-understanding of relevant ACP terminologies, the plan to learn more about ACP in the future, perceived norms from significant others, if any, to legally execute ACP documents, and possible engagement in ACP discussions.

All interviews were digitally recorded, transcribed verbatim, and independently coded in ATLAS.ti 5.0 Software (Scientific Software Development GmbH, Berlin, Germany) by two palliative care nurse reviewers [45,46]. A traditional thematic method was employed for qualitative data content analysis [38,47]. First, two coders individually categorized the data and created initial codes according to the semi-structured guide; data were reduced into visual displays of repeated words, patterns, clusters of phrases to indicate participants’ awareness, intention, and readiness to answer to our research questions. After the first two case interviews, code analysts met to compare their codes and discuss the direction of further analysis; the interview guide was again slightly modified according to two coders’ consensus. This deductive, iterative cycle [48] was to ensure congruence between the two coders regarding the study aims, data collection and analysis, until data saturation occurred. After interviewing nine cases, data saturation was determined while no new codes emerged, and the emerged codes were grouped to form a higher level of themes aligned with the Transtheoretical Model. We were confident that the final sample size of nine cases was sufficient to meet the purpose of multiple case exploration [29].

When findings were finalized for interpretations, the interdisciplinary team who helped to develop the questionnaire and the interview guide confirmed the study aims with respect to our interpretations of the data by re-examining the survey responses, transcripts, the list of emerged codes, and the overarching themes of TTM constructs: ACP awareness, intention, and readiness [49]. This process of systematically double-checking codes and creating a research decision trail would establish rigor and greatly enhance the credibility of our mixed-method data [48,50]. Reported in the following results section are a combination of quantitative descriptions and qualitative themes about terminal patients’ perceptions of behavioral awareness, intention, and readiness for ACP, under the theoretical guidance of the Transtheoretical Model.

### 2.4. Study Ethics

Our study was conducted from January to September 2017, after being approved by the Human Research Ethics Committee (#15MMHIS107) of the participating teaching hospitals and under rigorous supervision of the National Council of Indigenous Peoples in Taiwan. The final sample of older, community-dwelling indigenous patients with late-stage cancers, including their family caregivers in the data collection site, were provided with written documents and verbal explanations about the purpose, procedures, and contact information of this study. Since the risks for participating in this study were no greater than no participation, study subjects were assured that all survey and interview data, accessible only by the research team for five years, would remain confidential in all subsequent presentations and/or publications. In addition, cases’ survey and interview response were separated from their medical charts in the oncology clinic/hospital database. This nature of anonymous and voluntary participation was clearly explained before obtaining minority patients’ written informed consents.

## 3. Results

### 3.1. Study Participants

Despite specific efforts during study recruitment to obtain a diverse sample, older indigenous patients seemed to be homogenous in their age, gender, personal income, levels of education, and religious beliefs. The final sample was comprised of nine older (75.11 ± 3.85 years) patients (*n* = 9) from five diverse tribes with a variety of late-stage cancers (Table 1).

### 3.2. Awareness of Advance Care Planning

Our results indicated that despite all older indigenous patients being comfortable with discussing their own EOL, they never had the chance to do so before participating in this study. All patients were predominantly unaware of concepts related to ACP (Table 2), and none of the cases, nor their family caregivers, had prior experience making life-sustaining treatment (LST) decisions themselves. Older patients generally responded, “*These things never come up to me!*”; “*No one in the hospital told me about (ACP); they probably discussed with my daughter*”. Only one subject (74-year-old housewife with a diagnosis of stage III stomach cancer) who had an elementary school diploma was exposed to the term, but her understanding about the various LST options was very limited. “*I only heard about the term from a (oncology) nurse; I don’t know much about it!*”

The general understanding of LST was superficial, but cardiopulmonary resuscitations (CPR) and incubations (“tube-insertion”) for mechanical ventilators were allegedly the most recognized LST options cases often heard of from public media, not health care providers. Those more aware (*n* = 4, 44%) shared, “*I have heard about CPR, and (to be) hooked into the machine. They are things to keep the dying alive*”.

All patients with late-stage cancers initially had difficulties comprehending the purpose, types, or options of LST. During the study, our indigenous cases, mostly Christians, had concerns about prolonging life at the EOL. LST was regarded as “*a new and complicated idea that is, nonetheless, against nature,*” and initiating LST brazenly challenges God’s authority to predetermine the length of life. It was unsurprising that ACP was foreign to our indigenous cases with late-stage cancers. Executing advance directives (ADs), Durable Power of Attorneys (DOPAs) or initiating ACP were complicated ideas, and our patients showed relative impatience while learning these concepts.

Before participating in the study, none of the cases had heard about do-not-resuscitate (DNR) orders or ADs, and all (100%) were unable to discern financial wills from AD documents, such as living wills, and durable power of attorneys for health care (DOPAs). Only one 76-year-old male subject recalled that he might have heard “*something about planning for your own death in advance*” during his hospitalization from his physician. However, this patient’s understanding was incorrect: “*This planning is for my* *farewell ceremony…NO! I do not know ACP is about my own treatment or care on my death bed*”.

### 3.3. Advance Care Planning Behavioral Intention and Readiness for EOL Communication

According to the TTM, the entire group of older indigenous subjects was at a fairly low readiness stage for ACP behavioral change—due to little ACP awareness and no prior experience of ADs/DOPAs completion or any discussions related to LST or EOL care with physicians or significant others. The closest ACP conversations would be, self-proclaimed statements, casually mentioned to family and friends, to avoid futile treatment (“*Don’t resuscitate me if I am hopeless!*”). Such LST value revelations or declarations were frequently triggered by TV scenes or friends’ deaths. Our cases were collectively classified as precontemplator-believers (100%) [5] (Table 3) and benefits of “planning in advance” were commonly recognized, “*planning ahead is always good, especially for important matters related to life and death*”.

However, despite the majority (*n* = 66.66%) expressing a desire for dignified deaths, lack of intention to initiate ACP was observed. After ACP was explained in the interviews, only 44% of the sample (*n* = 4), the more educated who had no family caregivers, were able to complete own ADs, “*I can surely write this down. I want no pain and no sufferings (at the end)*”. For the rest of the lower educated indigenous patients, over and above unawareness, their ACP readiness remained low when first exposed to ACP. They were perplexed as to why advanced communication leaded to better EOL outcomes. “*I need time to process this idea… telling others about my wish…would it make (my dying) any difference?*”; “*No need to talk. I will die when the time comes*”. In addition, indigenous Christians believed that the timing of death was predestined and in God’s hands, and life, as a gift from God, should not be planned.

While EOL communication within the family had low priority in addition to insufficient ACP literacy, the majority of indigenous patients trusted that their physicians as medical experts would execute their professional responsibilities at their EOL. Two terminal patients (22.22%) even expressed that they passively waited for their physicians to use all possible modern technology to ultimately prolong their lives. “*They (doctors) surely will do their best (to prolong my life); oh yes, as long as possible*”. “*No need for me to plan too much. It doesn’t matter whether we discuss with families or not*”.

Before participating in this study, being entitled to make autonomous decisions was not paramount to our subjects. After learning about the goal of ACP, as a value exchanging process with loved ones to express EOL preferences, indigenous patients with late-stage cancers agreed that it would be worthwhile to re-evaluate life-prolonging process and communicate with others in advance, “*I want to tell them I have lived long enough. Don’t try too hard (to save me)*”. During the interview, patients gradually realized that ACP was to preserve patient autonomy under the circumstances to forgo futile, aggressive treatment for a dignified, peaceful death (*n* = 9, 100%). “*Maybe telling them what I want...I wanna die with peace. I can go see God now. Death is not terrible and life goes back to its natural cycle!*” While the majority had no caregivers, terminal indigenous patients had little normative pressure from significant others; they either required no approvals or had no one to discuss with (*n* = 8, 88.88%).

## 4. Discussion

### 4.1. Disparity Factors Influence Advance Care Planning Awareness in Ethnically and Geographically Minority Patients

Health disparities in quality EOL care exist across all racial and ethnic minority groups [34]. Our subjects’ socio-demographic characteristics fit into the typical profile of a disadvantaged group: ethnically minority patients who reside in underserved, remote areas are predominantly self-cared, spouseless, unemployed, and low-educated with little income (Table 1). In Euro-American countries, ACP uptake ranges from as high as 60%, mostly among white and well-educated patients, to as low as under 5% in minority cases [9]. The lack of relevant ACP knowledge has been documented as the major barrier in underserved populations [51,52], and in this present study, the general unawareness of our indigenous cases explains their extremely low to zero ACP uptake [13], little intention of behavioral change, and low readiness to engage in ACP.

We also found that our older patients with life-threatening illnesses never used any local hospice service nor received appropriate palliative care in remote community settings. After analyses conducted within cases and across cases, we conclude that our participants’ minimal experience with health care systems, little exposure to care resources, and limited access to mainstream information have jointly contributed to uninformed EOL options, including options available at the EOL [51]. With such disparities, it becomes difficult for treatment and care conversations to occur naturally between these inexperienced patients and their significant others.

It is worth mentioning that the issue of distrust of the health care system common in other minority patients [53] does not seem to be prevalent among our older indigenous participants; they expressed a fairly high level of confidence and satisfaction regarding their physicians’ treatment capability and nurses’ care quality under Taiwan’s comprehensive national health insurance. This fundamental trust of health care professionals and systems could be strategically utilized in promoting ACP. In addition, nurses in oncology wards may be a good source for providing relevant ACP information. However, health care professionals caring for minority cancer patients in underserved areas require extra support and additional resources allocated to create opportunities to initiate meaningful LST and EOL care discussions [54].

Unawareness (never heard about ACP) was the major ACP inhibitor among patients with cancer, and various types of ACP knowledge deficits could be characterized as: limited functional literacy (understanding of EOL language), lack of interactive literacy (opportunities for meaningful EOL discussion with providers and families), and insufficient critical literacy (uncertainty about future care) [9]. Spelten and colleagues suggest strategies for disparities and shared decision-making being incorporated in a tailored approach to improve ACP uptakes. After fully exploring our minority patients’ ACP awareness, participants exhibited no critical literacy but severe deficits of functional and interactive types of ACP literacy. Therefore, during our qualitative interviews, an introductory 20–30 min ACP session was offered by the interviewer with hopes of increasing subjects’ understanding. Information was provided concerning the purpose of engaging with ACP, explanations of relevant terminologies, and know-how concerning legal documents, such as ADs and DOPAs, and directions to nearby ACP clinics, etc.

Unfortunately, our pilot educational endeavors did not seem to promptly increase participants’ ACP knowledge about LST, ADs, and ACP. Merely being aware of ACP as a health promotion means only slightly motivates some patients to contemplate a dignified death. Although the idea of implementing patient autonomy is well-accepted by most patients without caregivers, this endorsement is not sufficient for patients to increase their intention to immediately engage in EOL communication. In other words, participants’ newly obtained ACP functional literacy [5] does not promptly facilitate intentions to initiate ACP, and, without interactive literacy, it is difficult for minority patients with little ACP knowledge to legally execute ADs or DOPAs.

What we learned from unaware minority patients’ ACP uptake was that a brief ACP introduction within 30 min, including explanations of definitions and/or terminologies with relevant pamphlets, remains insufficient to elevate readiness. What need be strategically incorporated into educational interventions are real-life LST scenarios so that the dying and their caregivers may take time to ponder and exchange life values. Ongoing counseling offered by health care providers during oncology or palliative care clinic visits may be essential to effectively overcome individual knowledge deficiencies to realize ACP as an imperative option at the EOL, particularly increasing minority patients’ willingness to initiate and maintain EOL discussions with significant others, if any. Transitioning precontemplator-believers to contemplators also requires culturally appropriate assistance. Continuous efforts from local health care providers who are cultural insiders that gradually break minority patients’ multiple barriers to learning information, accessing resources, and implementing ACP decision-making will be key to achieve ultimate EOL outcomes. In addition, although our older indigenous patients are mostly self-cared for and affected little by the opinions of their significant others, their ACP would remain indefinite if intervention strategies target only the knowledge domain at the individual level without considering other normative beliefs, such as unique cultural and religious influences in each tribe. For example, palliative care and advanced plans for dying at home have been highly accepted by the Dawu tribe on Orchid Island for their cultural need to bury the deceased within 24 h [55].

### 4.2. Christian Religiosity and Advance Care Planning Autonomy

While indigenous people’s tribal traditions have very little influence on our subjects’ spirituality, the majority of our sample, identifying parents’ religious beliefs, claimed themselves to be “*fairly religious*” Catholics or Protestant Christians (*n* = 4, 55.55%). This inclination is consistent with an early cohort report that approximately 70% indigenous Taiwanese profess Christian faith [44]. Although the influence of religions and spirituality on ACP has not been consistently discussed [56], among various ethnic patients who have advanced cancers, high levels of religiosity are found to associate with low ACP uptakes; religious patients tend to use aggressive treatment and have more deaths in the intensive care unit (ICU) [57]. Literature also suggests that the more religious the oncology patients are, the more likely they are to prefer life-prolonging care and rarely appoint DOPAs [58]. This may be because religious patients usually have hopes for miracles, trust in God’s will, and believe in fatalism, leading to a passive wish for family members, physicians, God, or other people to make EOL decisions on their behalf [34]. We observe this similarly in minority patients in that the more religious they self-report as, the more likely they tend to be to forgo LST and allow nature or God to take its course.

In our small sample, Christian identity and religiosity seem to serve as a barrier for older indigenous patients to contemplate LST and engage in ACP. Only one participant who happens to have no religious preference expressed the desire to initiate ACP within 6 months (11.11%); in addition to a prevalent fatalistic attitude toward their cancer disease [59], God’s sovereignty is overt among the indigenous Christians (*n* = 4, 44.44%). This religious conviction is also prevailing among Chinese-American Christian immigrants [40], that God’s plan takes priority over human autonomy and potentially leads to ACP procrastinations [41]. However, since a pursuit of peaceful, dignified deaths holds true universally, the desire, “*to leave the world well*”, may be strategized in ACP counseling so that older minority patients with late-cancers may further contemplate their own quality of dying. The evidence thereafter is that one participant explicates his goal of ACP as “*speaking my final wishes out loud to prevent miserable deaths*”. Our triangulation findings highlight the significance of religiosity, fatalistic beliefs, and life-support values in ACP decision-making.

In addition, in our findings the preference of patient autonomy in EOL decision-making is prominent, which is different from the Taiwanese mainstream culture. The shared decision approach favored by most older Taiwanese that LST consensus is reached among family caregivers [5,60] is surprisingly not endorsed by our older indigenous cancer survivors. We believe that this individualistic inclination, that EOL decisions ought to be made by terminal patients themselves irrespective of significant others’ influence, is related to our minority subjects’ unique family-less status and possibly to the basis of their religiosity, which varies from the folklore Taoism and traditional Buddhism popular in Taiwan [40,61]. Further investigation into incongruent decision-making discrepancies at EOL is imperative among diverse Taiwanese to better support patient autonomy in various populations. Common strategies in most Asian cultures that EOL discussions be facilitated within the family context [10,62] may not appeal to certain ethnically minority patients who highly value independence and/or are without EOL caregivers.

### 4.3. Study Strengths and Limitations

Up to now, Taiwanese studies related to indigenous peoples’ death and dying have been limited to epidemiological reports [18,63,64], funeral rituals [65,66], and local surveys [17,64]. Only one focus group has attempted to address healthy indigenous teenagers’ cultural view of death [67], and another qualitative study which explored Zhou tribes’ dilemmas around “a good death” [19]. Neither of them included any ACP elements. Current national cancer studies’ results were based entirely on the majority of non-indigenous, general residents, yet they constituted less than 1% of the mountainous indigenous cohort [15]. Reported percentages of cancer cohorts have been too small to examine ACP readiness across all indigenous peoples. Although there were a few indigenous workshops and some community studies held during the national ACP campaigns [60,68,69,70] to introduce the concepts of EOL decision-making and palliative care for this cultural group, it is still unclear to what extent these findings could be generalized beyond the majority of Taiwanese (Han tribe) in urban areas.

The sequential triangulation design [71] employed in this study permits in-depth exploration of ACP readiness among underserved patients of limited life expectancy. Our study uses the TTM as a framework and attempts to ensure methodological coherence between the study design and research inquiry; in particular, the sample is selected representative of our research topic. In order to explore ACP readiness as a complex behavior of health promotion, the results from multiple data sources are combined to present ACP behavioral precursors of awareness and intention, deviant from non-indigenous older patients in the mainstream society. We are able to reveal that in addition to those widely documented factors of health disparities, such as socioeconomic status, health literacy, language barriers, access barriers, lack of health insurance, lack of health care funding, and difficulty navigating health care systems and cancer networks [34,41], patients’ equipped knowledge and underlying religious backgrounds have fundamentally influenced discrepancies in EOL decision-making. While the preference of patient autonomy is highly related to the status of family caregiver, continuing research in older patients with late-stage cancers is required across various indigenous communities in different parts of Taiwan.

Although our multiple-case approach allows for a more comprehensive exploration and theoretical examination of the specific ACP phenomenon arising from a particular entity [2,40], the multiple limitations embedded in this study design must be acknowledged. Our study participants are from indigenous communities in eastern Taiwan only; they mostly reside in mountainous, less populated, and difficult-to-reach areas. Our findings represent neither older patients’ perspectives in suburban and metropolitan settings nor indigenous groups in northern or western Taiwan. The compromised generalizability by the potential selection bias of our relatively small sample must also be recognized, as it focuses solely on heterogeneous patients of multiple indigenous tribes with only certain types of late-stage cancers from a geographically restricted area. Our staff members in participating clinics have played a vital role in locating and approaching qualified patients; they assess patient criteria, follow initial contacts, and encourage their indigenous friends and relatives who may also be potential participants. Future researchers who are interested in recruiting difficult-to-reach indigenous patients with life-threatening illnesses, considering the sensitive nature of such studies, may use a snowball or networking method to conveniently recruit subjects [72], however, we do suggest increasing the variety of subjects in a larger sample.

## 5. Conclusions

This cross-sectional, multiple-case study is by far the first mixed-method study that explores ethnically minority patients’ complex ACP behavior in Taiwan. We found that older terminal indigenous patients facing imminent deaths have demonstrated great potential to be aware of ACP, comprehend LST, participate in EOL conversations, and complete their own ADs. However, ACP readiness requires time and continuous facilitations even among precontemplator-believers. We suggest counseling sessions be initiated in early cancer stages to eliminate significant knowledge barriers unique to minority patients with health disparities. By incorporating specific religious values, Christianity in this case, a culturally and spiritually competent ACP model may be better accepted if it attends to fatalistic beliefs, fosters spiritual care at the EOL, and achieves dignified deaths by underserved indigenous patients, since their desired EOL outcome is to avoid futile treatment and unnecessary sufferings [57]. In particular, information specifically tailored for patients with various types of knowledge barriers and without family caregivers may greatly decrease literacy discrepancies and ensure EOL care consistent with minority patients’ preferences. This Taiwanese indigenous study calls attention to an understudied area of ACP behaviors, an overlooked need in EOL care for older cancer patients of unique cultural and religious backgrounds, and the imperativeness to ensure culturally minority group’s EOL care is consistent with patients’ preferences.

## Figures and Tables

**Table 1:** Demographical characteristics of the older indigenous sample (*n* = 9).

Characteristics		Ratio
Age		
(Mean = 75.11 ± 3.86)	70–74 years old	6/9
	75–79 years old	2/9
	80–84 years old	1/9
Tribes		
	Payuan	2/9
	Amis/Pangcah	3/9
	Atayal	1/9
	Puyuma	2/9
	Bunun	1/9
Gender		
	Male	6/9
	Female	3/9
Origins of malignant neoplasm	Liver (Hepatoma) T4N1M1	2/9
	Hypopharynx T4aN3M0	1/9
	Stomach T4bN3aM0 & T4bN3bM1	2/9
	Lung T4N2M1b	1/9
	Colon/Rectum T4aN2aM1	1/9
	Ampulla of water T3bN2M1recurrent	1/9
	Duodenum T4N0M1	1/9
Primary Caregiver		
	No one (Self-cared)	6/9
	Family caregiver(s)	2/9
	Friend caretaker	1/9
Marital status		
	Married/partnered	4/9
	Widowed	5/9
	Divorced/separated	0
Highest education		
	Uneducated	2/9
	Some with no diploma	3/9
	Elementary school	4/9
Employment		
	No job	6/9
	Part-time	1/9
	Housewife	1/9
	(Tenant) Farmer	1/9
Personal income per month ($1NTD = $0.3USD)		
	No income at all	2/9
	<15,000 NTD (<$500 USD)	5/9
	15,000~33,000 NTD ($500–$1100 USD)	2/9
Raised beliefs		
	No tribal influence	2/9
	Mother’s tribal spirit	1/9
	Father’s tribal spirit	2/9
	Catholic/Christianity	4/9
Current beliefs		
	Atheist	0/9
	No religious preference	2/9
	Buddhist	2/9
	Taoist/Taiwanese	1/9
	Catholic/Protestant	4/9

**Table 2:** Advance care planning awareness of the sample (*n* = 9).

Questions	*n*	%
BEFORE TODAY, have you ever heard this term “life-sustaining treatment?”		
- Never	8	88.89
- Yes, I have (“*from an oncology nurse*”).	1	11.11
2 How much do you think you understand what “life-sustaining treatment” is?		
- Not at all	8	88.89
- A little bit (“*I only heard the term, nothing more.*”)	1	11.11
3 Before today, have you ever heard this term “Advance directives (ADs)? ”		
- No. Never.	9	100
4 Before today, have you ever heard this term, “Durable Power of Attorneys for Health Care (DPOAs)?”		
- No. Never.	9	100
5 How much do you think you understand the legal documents of “advance directives” and “durable power of attorneys for health care?”		
- Not at all	9	100
6 Before today, have you ever heard this term, “Advance Care Planning (ACP)?”		
- No. Never.	8	88.89
- A little bit (“*Something about planning death in advance, maybe?*”)	1	11.11
7 How much do you think you understand the concept of ACP in Taiwan?		
- Not at all (“*I just learned from you that this planning is related to treatment and care before I die.*”)	9	100

**Table 3:** ACP Behavioral Engagement of Older Indigenous Patients with Late-stage Cancers in Taiwan (*n* = 9).

Survey Questions	Precontemplation (Non-Believer)	Precontemplation (Believer)
1. Are you ready to plan for your future life-sustaining treatment and possible care at your death bed?	No, I don’t need to plan (0%).	- I don’t know if I should plan (*n* = 4; 44.44%)
		- I am ready to plan, but I don’t know when (*n* = 4; 44.44%)
		- I am ready to plan for my treatment and care within 6 months (*n* = 0%)
		- Yes, I am willing to plan but probably later (after 6 months) (*n* = 1; 11.11%)
2. Do you want to know more about “Advance care planning (ACP)?”	No, I don’t need to know more (*n* = 1; 11.11%).	- Maybe. I am not sure, since I just heard about ACP today (*n* = 4; 44.44%) - I want to know more about ACP, but I don’t know when to start (*n* = 3; 33.33%)
		- Yes, I want to know more about ACP, but NOT now (after 6 months) (0%)
		- Yes, I want to know more about ACP, soon within 6 months (1 = 1; 11.11%)
3. Do you think it is necessary now to plan for your future life-sustaining treatment and possible care at your death bed?	No, no need. (0%)	- Yes, it is of course necessary to discuss this now (*n* = 4; 44.44%)
		- Maybe, I don’t know if it is necessary to do so now (*n* = 5; 55.55%)
4. Have you signed ANY advance directive (ADs) or appointed any durable power of attorneys for health care (DPOAs)?		- No (*n* = 9; 100%)
5. If you have not signed an advance directive, are you ABLE to sign one today?		- Yes, I can complete one right now. (*n* = 4; 44.44%)
		- I don’t know. I need to know more first. (*n* = 3; 33.33%)
		- No, I cannot (*n* = 2; 22.22%) (I do not want to commit to anything)
6. Do you need to get someone’s approval before signing a document about your future treatment and possible care at your end-of-life??		- Yes, I need to be approved (0%)
		- Maybe, I am not sure. I have not thought about this before (*n* = 3, 33.33%).
		- No, I don’t need approvals from others (*n* = 6, 66.66%)
7. Do you prefer to discuss with others before signing a document about your future treatment and possible care at the end-of-life?		- Yes, I’d like to discuss (with my daughter) (*n* = 1; 11.11%)
		- No, I have no one to discuss with (*n* = 3; 33.33%)
		- I don’t know if I need to discuss with anyone (*n* = 2; 22.22%)
		- No, I don’t need to discuss with anyone (*n* = 3; 33.33%)

## Data Availability

The data presented in this study are available on request from the corresponding author. The data are not publicly available due to current funding restrictions and privacy and protection of indigenous patients.
